# A Comparative Study on the Effect of Amiodarone and Metaprolol for Prevention of Arrythmias after Open Heart Surgery

**Published:** 2013-03-15

**Authors:** Nayeem‑ul‑hassan, Abdul Majeed Dar, Mohd Lateef Wani, Hilal Ahmad Rather, Farooq Ahmad Ganie

**Affiliations:** 1 Department of Cardiovascular and Thoracic Surgery, Sher-i- Kashmir Institute of Medical Sciences, Soura Srinagar, Kashmir, India; 2 Department of Cardiology, Sher-i- Kashmir Institute of Medical Sciences, Soura Srinagar, Kashmir, India

**Keywords:** Amiodarone, Metaprolol, Atrial Fibrillation

## Abstract

**Objectives:**

The aim of this study was to compare the effect of amiodarone and metaprolol in prevention of atrial fibrillation in patients, following open heart surgery.

**Methods:**

This prospective study was carried out between May 2008 to Nov. 2010, and comprised a total of 50 patients with normal preoperative sinus rhythm undergoing open heart surgery using cardio pulmonary bypass.

**Results:**

Mean age of patients was 47+2.7 years, of which 60% who developed atrial fibrillation aged from 51 to 60 years. Most patients (62%) were in NYHA Class III. Patients who received amiodarone showed significant improvement in LVEF compared to those treated with Metaprolol. Amiodarone treated group exhibited lesser incidence and short-lasting atrial fibrillation, lower ventricular rate, shorter hospitalization, and lesser cost of care than those in metaprolol group.

**Conclusions:**

The present study showed that amiodarone was more efficient in controlling post-operative atrial fibrillation as compared to metaprolol. However, a larger randomized controlled trial is needed to corroborate the result of this study.

## 1. Background

Postoperative cardiac arrhythmias are a frequent complication of open heart operations. Supraventricular tachyarrhythmia (SVT), particularly atrial fibrillation (AF), is the most common arrhythmia developing after operation in these patients. In an analysis of 8 large cardiac surgery trials totaling 20,193 patients, the incidence of postoperative AF was estimated to be 26% and ranged from 17% to 35%,(1). Even though postoperative AF is often short-lived with self-limiting complication accompanied by increasing morbidity. The incidence of postoperative stroke, the length of stay in hospital (LOS), the incidence of ventricular arrhythmias, and the need for a permanent pacemaker are all increased in patients who develop AF after cardiac operations(2-4). Thus, to improve the care of patients undergoing open heart operations, effective prophylactic measures should be taken to prevent postoperative AF. The present study was then undertaken to assess the efficiency of amiadarone compared to metaprolol in preventing post operative AF.

## 2. Patients and Methods

This prospective study was conducted in the epartment of Cardiovascular and Thoracic Surgery and Cardiology Sher-i-Kashmir Institute of Medical Sciences over period of 30 months from May 2008 to Nov. 2010. The study comprised a total of 50 patients with normal preoperative sinus rhythm undergoing open heart surgery on cardio pulmonary bypass. 

A demographic record of the patients was made which included name, age, sex address, occupation, marital status and a number of other issues. The patient’ detailed history was then taken which included breathlessness, palpitations, cough, hemoptysis, chest pain, syncope, recurrent chest infections, fatigue as well as those relevant to cardiovascular and thoracic system. Notes were also taken of the history of rheumatic fever, joint pain and swelling, diabetes mellitus, hypertension ,and rheumatic prophylaxis. Every patient was evaluated in regard to all symptoms and assigned a functional class according to the guideline of New York Heart Association (NYHA) classification. A detailed clinical examination was carried out with special references to cardiovascular system. Information about other organ systems was obtained to rule out any medical co-morbidity in order to minimize the selection bias. Also further studies of the patients were carried out to exclude other organ system dysfunction, so that the patients under study were free of any confounding factors. A current protocol for open heart surgery was followed to prepare the patients for operation. The patients’ consent were obtained after briefing them on the nature of surgery.

Patients were then randomized into amiodarone and metaprolol groups. The amiodarone group were put on calculated dose of oral amiodarone 10mg per kg body weight, two weeks before surgery and continued till hospital discharge.

The metaprolol group were put on calculated dose of oral metaprolol ( 25mg 8 hourly for heart rate 60-70 beats per minute, 50mg 12 hourly for heart rate 70-80 beats per minute, 50mg 8 hourly for heart rate more than 80 beats per minute) two weeks before surgery and continued till hospital discharge. 

The episodes of atrial fibrillation were counted, if continued more than 5 minutes during or after open heart surgery until hospital discharge.

Intra-operatively electrocardiogram was monitored throughout the procedure. Surgical procedure was carried out through median sternotomy or right thoracotomy.Blood gas analysis, electrolytes and activated clotting time were continuously monitored throughout surgical operation, as well as recording the cardiopulmonary bypass and cross clamp times, type of valve, its size and position. The patients were transferred to surgical intensive care unit. As soon as patients’ condition allowed, they were shifted to cardiovascular and thoracic ward. Serum potassium was kept above 4 meq/ liter all along.

Immediately after operation, all patients were monitored in the intensive care unit. During this time all arrhythmias were recorded on rhythm strip and documented in the bedside chart. When transferred to the Ward, rhythm monitoring was continued. The post operative results were analyzed as follows:

Incidence of atrial fibrillation in amiodarone and metaprolol group. Timing of development of atrial fibrillation.

Duration of atrial fibrillation: Maximum ventricular rate during atrial fibrillation. 

Spontaneous conversion rate: Hospitalization period and its associated costs. Atrial fibrillation associated risk factors: 

### 

## 2.1 Statistical Analysis

Data was compiled and analysed statistically by using SPSS soft ware. Fishers Exact T Test was used.

## 3. Results

This study was conducted during May 2008 to Nov. 2010, and comprised 50 patients with rheumatic valvular heart disease and congenital heart disorders with normal sinus rhythm. The patients underwent correction of congenital cardiac defects, mitral valve replacement, aortic valve replacement and double valve replacement, under cardiopulmonary bypass using normothermic cardioplegia in Sher­i­ Kashmir Institute of Medical Sciences. Most patients aged from 41 to 50 years (mean Age 47±2.7 years). Male to female ratio was 1.5:1. Baseline characteristics of two groups of patients are given in Table 1. Atrial fibrillation developed in 60% of patients aged between 51-60 years, and 62% of patients were in NYHA Class III. On pre-operative Echocardiography mean left atrial (LA), left ventricular end diastolic dimension (LVEDD), left ventricular end systolic dimension (LVESD) and pulmonary arterial hypertension (PAH) were 45±3.31mm, 57±4.43 mm, 42.31±8.39 mm and 38±3.32 mmHg respectively. Also 33.33% of patients with AF had cardiothoracic ratio on chest X-ray (CTR) between 65-­70%. Patients on amiodarone showed significant improvement in LVEF compared to Metaprolol (Table 2). Maximum number of patients (32%) had Mitral valve disease and had undergone valve replacement. Postoperatively, 25% of Mitral valve Replacemnt(MVR), 22.22% of Aortic Valve Replacement (AVR), 42.85% of Double Valve Replacement (DVR) , 16.66% of Atrial Septal Defect (ASD) and Ventricular Septal Defect (VSD) repair patients develop AF. In total, 12 patients (24%) develop AF postoperatively. 28.12% of valve replacement patients , compared to 16.66% of septal defect repair patients were complicated by postoperative AF(*P ≤ *0.05) artial fibrillation, AF of shorter duration, had lesser ventricular rate, shorter hospital stay, and lower cost of care compared to metaprolol group (Table 3). Among two groups AF was delayed in Amiodarone group ( 50% of AF >48 hours) ,compared to Metaprolol (50% AF 24-48 hours ) In amiodarone group, a case of sudden death occurred postoperatively compared to two deaths in metaprolol group during the hospital stay which was only due to cardiac reasons. Other postoperative complications were acceptable excepting 24% of patients with ARF (Acute Renal Failure) with insignificant statistical difference between two groups (Table 4). 

**Table 1.** Baseline Characteristics of Patients in Two Groups

Characteristic	Amiodarone	Metaprolol	*P value*
Age	46+ 2.3 years	48+ 2.7years	NS
Male:Female ratio	1.5:1	1.5:1	NS
NHYA[Table-fn fn4927] grade	2.46+ 1.10	2.52 +0.97	NS
Prior MI[Table-fn fn4927]	10%	10%	NS
COPD[Table-fn fn4927]	5%	6.66%	NS
CHF[Table-fn fn4927]	15%	16.66%	NS
HT[Table-fn fn4927]	35%	36.66%	NS
DM[Table-fn fn4927]	10%	10%	NS
Beta blocker withdrawal	None	None	NS
LVSF[Table-fn fn4927]%	40.2 +12.2	38.9+ 8.2	NS
LVEF[Table-fn fn4927]%	58.83+ 8.6	60.37+ 9.02	NS

Abbervations: NYHA, New York Heart Association; MI, Myocardial Infarction; COPD, Chronic obstructive pulmonary disease; CHF, Congestive Heart Failure; HT, Hypertention; DM, Diabetes Milletus; LVSF, Left ventricular systolic function; LVEF, left ventricular ejection fraction.

**Table 2.** Left Ventricular Ejection Fraction(LVEF) in Two Groups

Group	Preoperative LVEF	Post Operative LVEF	*P Value*
Amiodarone Group	58.83+8.6	62.32+9.92	<0.05
Metaprolol Group	60.37+9.02	59.2+11.7	>0.05

**Table 3.** Postoperative Characteristics of Patients

Characteristic	Amiodarone n=20	Metaprolol n=30	*P value*
AF[Table-fn fn4928]%age	20%	26.7%	NS
Time of Development of AF	62.15+ 18	48.4+ 10.2	NS
Duration of AF	12.04+ 10.2	18.8+ 10.4	NS
Maximum Ventricular rate during AF	118.+ 30.4	148+ 22.2	NS
AF on discharge	1	3	NS
Length of hospital stay	12.7+ 4.5	13.2+ 5.2	NS
Cost of Care	1.17+ 0.12	1.18+0.10	NS

Patients in amiodarone group have less incidence of AF, lasts shorter, have lesser ventricular rate, shorter hospital stay, lesser cost of care compared to metaprolol group.

**Table 4.** Postoperative Complications

Complication	Amiodarone	Metaprolol	*P value*
Sudden death	1	2	NS
Ventricular arrhythmia	1	5	NS
Sinus bradycardia	2	3	NS
AV Block	2	3	NS
Hypotension	7	9	NS
GI Tract symptoms	5	0	0.016
Liver toxicity	1	0	NS
ARF	4	7	NS

## 4. Discussion

 Atrial fibrillation (AF) is the most common arrhythmia observed after cardiac operations with an incidence of 30–60%,(2, 5). Although postoperative mortality does not seem to be affected by this complication, AF can lead to hemodynamic compromise, carries the risk for thromboembolic events and frequently requires antiarrhythmic drugs,(5-7). Duration of ICU stay and overall hospitalization may be prolonged as a result of this complication, which contributes substantially to increased costs(2, 5). The incidence of AF after cardiac surgery is influenced by various factors such as type of cardiac surgery, age of the patient and perioperative treatment or withdrawing betablockers(5, 8), Age has been repeatedly shown to be the major risk factor for atrial fibrillation after cardiac surgery(5, 8), Our results show that for every 10‑­year increase in age, there is 75% increase in the odds of developing atrial fibrillation. Thus on the basis of age alone, subjects older than 70 years are considered to be at high risk for developing atrial fibrillation.

Researchers found that patients with a history of AF were at increasing risk for postoperative AF. Mathew(9), et al found that postoperative AF increased by approximately 2‑­fold in patients with a history of AF.

Also Song et al, found that age, history of AF, ejection fraction less than 50%, LA size more than 50 mm, aortic cross clamp time were significantly related to post open heart AF (10).

Our study showed that smoking, gender, preoperative pulmonary arterial hypertension, left ventricular and diastolic and end systolic dimensions were not significantly related to the development of postoperative AF. (*P*> 0.01).These results were consistant with Mathew et al. (9).

Also our study showed that amiodarone group of patients experienced less incidence of postoperative AF, delayed emergence of AF, duration of AF, slower ventricular response during AF, better postoperative ejection fraction,and shorter hospital stay compared to the patients on Metaprolol. All the patients tolerated amiodarone well with acceptable side effect profile. Postoperative LFT and thyroid function tests were within normal limits. There was no significant difference in other post operative complication rate and total hospital costs between two groups of patients.

According to the double blind randomized placebo controlled study the study of Song et al in(10), postoperative AF occurred in 16% of patients receiving amiodarone and in 37.7% of cases receiving placebo(*P*=0.006). AF occurred at 58.13±16.63 hours in low dose amiodarone group and at 45.03±17.0 hours in control group (*P*=0.018).The maximum ventricular rate during AF was significantly slower in the low dose amiodarone group (121.42±28.91beats/min) than in control group (134.11±30.57) beats/minute (*P*=0.036). The duration of AF was10.92±9.56 hours for low dose amiodarone group compared with 14.81±10.37 hours in placebo group (*P*=0.002). Postoperative LVEF was significantly improved in low dose amiodarone group compared to placebo. Low dose amiodarone group had shorter hospital stay and lower total hospital costs compared to placebo group.

Crystal et al carried out meta­‑analysis on 52 randomized trials and concluded that beta blockers reduced the percentage of patients with AF from 33% in control group to 19% in beta blocker group with no significant effect on hospital stay after open heart surgery(11). Additionally, percentage of patients with AF was reduced from 37% in control group to 22.2% in group with significant effect between length of hospital stay and total hospital costs.
